# A conserved bioelectrical signature defines subventricular zone-derived human fetal neural stem cells and tracks their differentiation state

**DOI:** 10.3389/fcell.2026.1774119

**Published:** 2026-03-10

**Authors:** Roberta De Zio, Diletta Lucia Capobianco, Daniela Celeste Profico, Giada D’Aloisio, Giuseppe Procino, Maurizio Gelati, Angelo Luigi Vescovi, Francesco Pisani, Maria Svelto, Andrea Gerbino

**Affiliations:** 1 Department of Biosciences, Biotechnologies and Environment, University of Bari “Aldo Moro”, Bari, Italy; 2 ProPharma LLC, Leiden, Netherlands; 3 IRCSS Casa Sollievo della Sofferenza, Unità Produttiva per Terapie Avanzate, San Giovanni Rotondo, Italy; 4 Fondazione IRCSS Istituto Neurologico “C.Besta”, Milan, Italy; 5 Faculty of Medicine, Link Campus University, Rome, Italy; 6 Neurology Group, Abu Dhabi Stem Cell Center, Abu Dhabi, United Arab Emirates

**Keywords:** ion channel, K+ currents, resting membrane potential, stem cell, stemness marker

## Abstract

**Introduction:**

Human fetal neural stem cells (hfNSCs) from the subventricular zone (SVZ) are employed in clinical trials for neurodegenerative diseases, yet their bioelectrical properties remain largely unexplored. Molecular markers alone do not reliably correlate with functional state, highlighting the need for complementary functional descriptors.

**Methods:**

We performed whole-cell patch-clamp recordings on hfNSCs from three independent SVZ donors (15–16 weeks of gestation) to characterize resting membrane potential (Vm) and voltage-gated currents, assessing inter-donor reproducibility and differentiation dynamics over 30 days *in vitro*.

**Results:**

hfNSCs exhibited a highly reproducible bioelectrical signature across all donors, characterized by a depolarized resting membrane potential (∼−30 mV), a non-excitable profile, and a stereotyped composition of outward K+ currents. The two current components were resolved using combined biophysical and pharmacological approaches, while Western blot analysis confirmed the expression of Kv4.2 and Kv1.1 channel subtypes functionally consistent with I_A_ and I_K_, respectively. Remarkably, inter-donor variability in bioelectrical parameters was minimal despite independent cell line derivation. During differentiation, Vm underwent rapid hyperpolarization within 24 h, representing the earliest detectable functional transition. I_A_ showed progressive reduction detectable as early as 24 h and more pronounced by day 15, while I_K_ remained stable throughout. By day 30, inward voltage-gated currents emerged in approximately 60% of cells, consistent with progression toward more differentiated neuro-glial electrophysiological states; however, cells remained non-excitable under our recording conditions. This late-stage divergence highlights heterogeneity in maturation trajectories and completes a temporally ordered sequence of electrical remodeling.

**Conclusion:**

SVZ-derived hfNSCs possess a reproducible bioelectrical signature within the 15–16-week gestational window across independent donors, supporting electrophysiological profiling as a quantitative functional benchmark for identity and standardization. During *in vitro* differentiation, Vm hyperpolarization and I_A_/I_K_ remodeling track early functional progression, whereas the late emergence of inward currents in only a subset of cells indicates increased heterogeneity at later stages. Overall, these findings support bioelectrical profiling as a quantitative functional biomarker with potential utility for standardization and quality assessment in hfNSC-based regenerative therapies.

## 1 Introduction

Human neural stem cells (NSCs) derived from the subventricular zone (SVZ) are currently employed in clinical trials for neurodegenerative diseases due to their capacity to differentiate into neurons, astrocytes, and oligodendrocytes, and to integrate functionally into host neural circuits (Profico et al., 2022; Mazzini et al., 2019; Shetty and Hattiangady, 2016; Verkerke et al., 2025; Derso et al., 2025). In addition to cell replacement, NSCs exert potent neuroprotective effects through the release of neurotrophic and immunomodulatory factors, including extracellular vesicles, which help preserve host neurons and counteract ongoing pathological processes. This mechanism, often referred to as the “paracrine hypothesis” or “bystander effect,” underscores the multifaceted role of NSCs in modulating the neural microenvironment and promoting endogenous repair (Profico et al., 2022). Consistent with this concept, we recently showed that human fetal NSCs (hfNSCs) also contribute to neuroprotection through direct cell-to-cell communication, specifically via the formation of nestin-positive tunnelling nanotubes (TNTs) that mediate the transfer of functional mitochondria (Capobianco et al., 2024). The complexity and dynamic nature of hfNSC mechanisms of action highlight the need for functional parameters capable of reflecting their physiological state and therapeutic potential.

Currently, NSC characterization relies primarily on molecular markers such as Nestin, Sox-2, Musashi-1, and CD133 (Pevny et al., 2010; Sugawara et al., 2002; Li, 2013; Okano et al., 2005). However, these markers can be retained in partially differentiated cells and do not necessarily correlate with self-renewal capacity or regenerative potential (Sun et al., 2009). This limitation emphasizes the need for complementary functional biomarkers that can provide an accurate assessment of stem cell state. Among such candidates, the cellular bioelectrical profile has emerged as a conserved functional signature and a reliable physiological indicator of stem cell identity across multiple systems. For instance, a minimal set of biophysical parameters can effectively identify and predict the function of mesenchymal stromal cells within mixed populations (Lee et al., 2014). The resting membrane potential (Vm) and the ionic conductances that shape it act as fundamental bioelectric regulators of stem cell behavior (Sundelacruz et al., 2009), typically marking the transition from a proliferative, undifferentiated state (depolarized Vm) to a differentiated phenotype (hyperpolarized Vm). This regulatory mechanism is conserved across species and cell types and is driven by the coordinated remodeling of ionic channels, particularly K+ channels.

Despite this recognized importance, a systematic electrophysiological characterization of hfNSCs derived specifically from the SVZ, a *bona fide* neurogenic niche and a clinically validated source (Zhang et al., 2025) for NSC production, is still lacking. Moreover, the reproducibility of the hfNSC bioelectrical profile across independent donors remains largely unexplored. Here, we address this gap by profiling SVZ-derived hfNSCs from three independent donors. Defining inter-donor consistency is essential for two reasons: (Profico et al., 2022): to evaluate the suitability of the bioelectrical profile as a functional marker, and (Mazzini et al., 2019) to investigate donor-related variability, offering preliminary insights into the challenges of standardization and quality assurance for stem cell–based therapies. Previous electrophysiological studies have described the remodeling of K^+^ currents, specifically, the transition from transient A-type (I_A_) to sustained delayed-rectifier (I_K_) currents, in human fetal neural progenitor cells derived from the midbrain (Schaarschmidt et al., 2009). However, these findings cannot be directly extrapolated to SVZ-derived hfNSCs, which originate from a distinct neurogenic niche with broader neuro-glial differentiation potential and greater translational relevance for cell and gene therapy applications. This distinction is biologically relevant, as regional heterogeneity of NSCs, even within the developing brain, profoundly influences both molecular and functional properties (Andreotti et al., 2019). Furthermore, it remains unclear how Vm dynamics temporally relate to ion channel remodeling during lineage progression.

To provide further insights into this field, the present study aimed to (i) define the electrophysiological profile of human fetal SVZ-derived hfNSCs, focusing on Vm and voltage-gated currents; (ii) assess inter-donor stability of these bioelectrical parameters; and (iii) determine how this profile evolves during *in vitro* differentiation. By establishing the temporal relationship between Vm modulation and ion channel remodeling, this work supports electrophysiological profiling as an early functional readout of hfNSC differentiation progression. Understanding the intrinsic bioelectrical properties of SVZ-derived hfNSCs is essential for two reasons. Firstly, it expands our knowledge of the biophysical properties underlying stemness and lineage commitment. Secondly, it establishes robust functional criteria for assessing the consistency and quality of SVZ-derived hfNSCs in stem cell-based therapies.

## 2 Materials and methods

### 2.1 Human neural stem cell culture and differentiation

hfNSC primary cultures were generated as previously described and correspond to cell populations already validated for stemness and neuroprotective properties (Capobianco et al., 2024; Mazzini et al., 2015). Tissue procurement was approved by the Ethical Committee of the Institute “Casa Sollievo della Sofferenza di San Pio da Pietrelcina” (IRCCS). All specimens were collected solely from fetuses following natural, spontaneous *in utero* death, and only after obtaining the mother’s informed, written consent. Fetal brain tissue specimens from the forebrain of three donors (indicated as Donor 1, Donor 2 and Donor 3) who died at the 15th and 16th gestational weeks were transferred to the Good Manufacturing Practice (GMP) facility immediately after collection under rigorous sterile conditions. The tissue was washed in Phosphate-buffered saline (PBS), supplemented with gentamicin (50 μg/mL), and mechanically dissociated by pipetting to obtain a single-cell suspension. The cells were then placed in culture at a density of 1 × 10^4^ cells/cm^2^ in a chemically defined medium supplemented with 20 ng/mL EGF and 10 ng/mL bFGF, in accordance with established expansion protocols (Gritti et al., 1999). The cultures were then maintained in a humidified incubator at 37 °C with 5% O_2_ and 5% CO_2_, where they proliferated in the form of free-floating clusters (neurospheres). After 7–10 days, the neurospheres were collected by centrifugation and mechanically dissociated by pipetting. The resulting single-cell suspension was quantified using a Bürker chamber and replated at the same initial density. This step was routinely repeated to ensure the continued culturing of the cells. During expansion, aliquots of neurospheres were cryopreserved in culture medium supplemented with 10% dimethylsulfoxide (DMSO), as previously described (Profico et al., 2022; Mazzini et al., 2015). For all experiments requiring adherent culture conditions, glass substrates were coated with Cultrex (PathClear, catalogue no. 3432-005-01, diluted 1:50 in hNSC medium) for 1 hour at 37 °C, as instructed by the manufacturer. The cells were plated at a density of 1 × 10^4^ cells/cm^2^ for the patch clamp and microscopy experiments and analyzed once they were well attached. This represented our initial hfNSC-undifferentiated experimental condition. The differentiation protocol was applied as previously described (Rosati et al., 2018). Neurospheres after mechanical dissociation were resuspended in the same culture medium used during their routine culturing but in the absence of EGF and in the presence of the sole bFGF. Cells were seeded on a cultrex layer (Cultrex® Basement Membrane Extract, Trevigen) and incubated at 5% O2, 5% CO_2_ and 37 °C for 3 days. Then culture media was replaced with DMEM/F12 supplemented with 2% FBS, w/o growth factors. The onset of differentiation was defined as the time point at which the cells were resuspended in EGF-free medium. Electrophysiological recordings were subsequently performed at defined stages of the differentiation process, specifically at 24 h, 15 days, and 30 days after induction.

### 2.2 hfNSCs growth assessment

To document proliferative capacity during expansion, hfNSC growth was assessed *in vitro* by quantifying viable cell number at defined time points up to 30 days. At each time point, neurospheres were collected, mechanically dissociated to a single-cell suspension, and cells were counted using a Bürker chamber after Trypan blue staining to exclude non-viable cells. Growth curves were generated by plotting the recovered cell number as a function of days in culture.

### 2.3 Confocal microscopy

For microscopy analysis, cells were fixed with 4% paraformaldehyde (PFA) for 10 min at room temperature (RT), using the culture medium as the vehicle for fixation. Cells were then washed with PBS and permeabilized with 0.3% Triton X-100 in PBS for 15 min. Non-specific antibody binding sites were blocked by incubating the samples with 3% Bovine Serum Albumin (BSA) in PBS for 30 min at RT. The samples were then incubated with the primary antibodies, diluted in 3% BSA in PBS, for 24 h at 4°. The primary antibodies and their respective working dilutions were: CD15/SSEA1 (MC480) Mouse mAb (Cell Signaling, Cat. No. 4744) at 1:500; Sox-2 (L1D6A2) Mouse mAb (Cell Signaling, Cat. No. 4900) at 1:400; Nestin (10C2) Mouse mAb (Cell Signaling, Cat. No. 33475) at 1:1000; and Musashi-1 Antibody (R&D Systems, Cat. No. AF2628) at 1:100. Following the primary antibody incubation, samples received multiple gentle washes with 3% BSA in PBS. Secondary antibody incubation was performed for 1 hour at RT using the same blocking solution. The appropriate, highly cross-adsorbed secondary antibodies, diluted 1:1000, were selected based on the primary antibody host species: Donkey anti-Mouse IgG (H + L) Alexa Fluor™ 488 (Thermo Fisher, Cat. No. A-21202) was used for the mouse primaries (CD15, Sox-2, and Nestin), and Donkey anti-Goat IgG (H + L) Alexa Fluor™ 546 (Thermo Fisher, Cat. No. A-11056) was used for the goat primary (Musashi-1). Additionally, following the secondary antibody washes, F-actin was stained by incubating the samples for 1 h at RT with AlexaFluor488-Phalloidin (1:500; Thermo Fisher, cat. #A12379) in PBS. Finally, after further washes in PBS, the samples were mounted in Vectashield antifade mounting medium (Vector Laboratories, Cat. No. H-1000-10). Confocal microscopy was performed using a Leica TCS SP5 microscope.

### 2.4 Electrophysiology

Biophysical analysis of hfNSCs was performed using whole-cell patch-clamp experiments on isolated cells. Recordings were made using an Olympus B51WI microscope and a Multiclamp 700B amplifier (Axon CNS-Molecular Devices, Sunnyvale, CA, United States), interfaced with an Axon Digidata 1500 (Axon Instruments-Molecular Devices, Sunnyvale, CA, United States). Currents were sampled at 10 kHz and low-pass filtered at 5 kHz. Borosilicate patch pipettes, with a tip resistance of 5–7 MΩ, were pulled using a P-1000 Pipette Puller (Sutter Instrument, Novato, CA, United States) and filled with an internal solution containing (in mM): 130 K-gluconate, 10 NaCl, 1 CaCl_2_, 1 EGTA, 10 HEPES, 2 ATP-Na_2_, 2 MgCl_2_, pH 7.2 (adjusted with KOH), and an osmolarity of 280 mOsm/kg.

Recordings were performed at room temperature on hfNSC cells plated on Cultrex®-coated coverslips (15 mm Ø), using an extracellular solution composed of (in mM): 140 NaCl, 2.8 KCl, 1 CaCl_2_, 0.01 EDTA, 10 HEPES, pH 7.2 (adjusted with NaOH), and an osmolarity of 283–284 mOsm/kg. Vm was measured in current-clamp mode (gap-free), without current injection, for 2 consecutive minutes. Depolarizing current steps (500 m duration, with 5 pA increments) were applied to assess the cells’ ability to generate action potentials. Voltage-dependent currents were elicited by 300 m depolarizing steps from −100 mV to +70 mV, in 10 mV increments, from a holding potential of −85 mV. Current amplitudes were measured at two time points during each depolarizing voltage pulse: 0–20 m (transient component, t. c.) and 280–300 m (sustained component, s. c.). This approach exploits the distinct activation kinetics of the two components, with the t. c. (I_A_-like) rapidly inactivating and the s. c. (delayed-rectifier K^+^ current, I_k_-like) remaining active throughout the depolarization. Peak current amplitudes were normalized to membrane capacitance (pA/pF) and plotted as current-voltage (I-V) relationships. Current amplitudes were normalized to their peak values and fitted with a Boltzmann function to determine the half-activation and half-inactivation voltages (V_1_/_2_) and the slope factor (K). Inactivation kinetics were analyzed at +50 mV using 300 m test steps, following 500 m hyperpolarizing pre-pulses from −100 mV to +40 mV in 10 mV increments. As for the activation kinetics, peak values of the t. c were measured between 0–20 m, and the s. c. was analyzed between 280–300 m. Current amplitudes were normalized to their respective peak values and fitted with a Boltzmann function to determine the half-inactivation voltage (V_1_/_2_) and slope factor (K). In order to assess their nature, the two currents components were separated both biophysically and pharmacologically. Throughout the manuscript, outward current components are initially described based on their kinetic properties as transient (t.c.) and sustained (s.c.) components. Following biophysical and pharmacological isolation, these components are subsequently referred to as the A-type K^+^ current (I_A_) and the delayed-rectifier K^+^ current (I_K_), respectively. Biophysical separation of I_k_ was achieved using a 500 m depolarizing pre-pulse (from a −85 mV holding potential) to −40 mV, which selectively inactivated the I_A_; I_K_ was then recorded during 300 m depolarizing steps from −100 mV to +70 mV in 10 mV increments. Pharmacological isolation of the two components was achieved by perfusing 100 µM 4-aminopyridine to block I_A_ immediately before recording, thereby isolating the sustained I_k_ component, which was recorded during 300 m depolarizing steps from −100 mV to +70 mV (with −85 mV holding potential). The transient I_A_ component was then calculated by subtraction. Biophysical maturation of hfNSCs was monitored up to 30 days, measuring Vm, cell excitability and voltage-gated currents at 24 h, 15 days, and 30 days post-differentiation. Data for inward currents at 30 days refer to experiments where detectable evoked inward currents were observed. All data are presented as mean ± SEM. AxoScope 10.4 (Molecular Devices, Sunnyvale, CA, United States) and pClamp 10.4 (Molecular Devices, Sunnyvale, CA, United States) were used for data acquisition and analysis. For all electrophysiological measurements, mean values across donors are reported in the main text, whereas donor-specific values and full statistical comparisons are provided in the corresponding figure legends.

### 2.5 Western blot analysis

Proteins expression was analyzed by Western blotting following standard procedures. Briefly, cells were lysed using RIPA buffer containing (in mM): 150 NaCl, 10 Tris/HCl, 1% Triton X-100, 0.1% SDS, 1% deoxycholate-Na, and 5 mM EDTA (pH 7.2), supplemented with protease and phosphatase inhibitors (in mM: 10 NaF, 100 sodium orthovanadate, 15 sodium pyrophosphate). Cells were then sonicated at 60% amplitude using a Vibra-Cell® sonicator (Sonics and Materials Inc.), and the lysate was centrifuged at 13,000 rpm for 30 min at 4 °C to pellet the membranes. Protein concentrations were determined using the Bradford protein assay. Proteins were denatured in 1× Laemmli Sample Buffer (Bio-Rad) containing 50 mM DTT and subjected to SDS-PAGE followed by Western blotting as described below. Protein samples were electrophoresed on 7.5% polyacrylamide SDS gels (Mini-PROTEAN TGX Stain-Free Precast Gels; Bio-Rad) and transferred onto a 0.2-µm PVDF membrane (Trans-Blot Turbo Mini 0.2 µm PVDF Transfer Packs #1704156; Bio-Rad) using the Trans-Blot Turbo Transfer System (Bio-Rad). The membrane was then blocked with 5% BSA in TBS-T for 1 h at room temperature to prevent non-specific binding. It was subsequently incubated overnight at 4 °C with the primary antibody specific to Kv1.1 and Kv4.2 (Anti- K^+^ Channel Kv1.1: Sigma-Aldrich, Catalog # MABN616; Anti- K^+^ Channel Kv4.2: Proteintech, Catalog # 21298-1-AP), diluted in TBS-T with 1% BSA, at a 1:1000 dilution. Following primary antibody incubation, the membrane was washed 3 times with TBS-T for 10 min each and then incubated for 1 h with the appropriate HRP-conjugated secondary antibody (1:5000). Protein bands were detected using chemiluminescence and images were captured using the ChemiDoc system. Band intensity was quantified using Image Lab software.

### 2.6 Statistical analysis

GraphPad Prism 6 was used for the statistical analysis and graph representation of the data. Data are given as mean ± standard error of the mean (SEM). Statistical analysis was performed using one- or two‐way ANOVA test or with Student’s t-test for unpaired data depending on the data set analyzed.

## 3 Results

### 3.1 Validation of hfNSC identity

**FIGURE 1:**
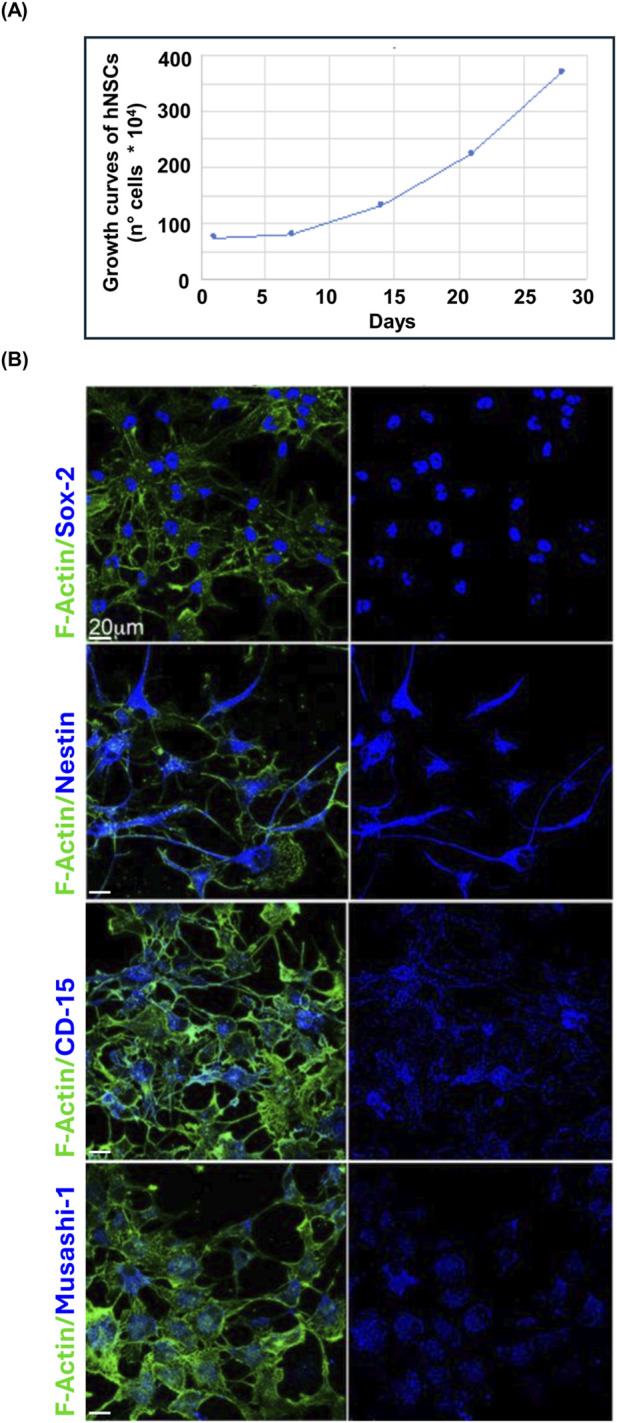
Confocal immunofluorescence analysis of hfNSCs. **(A)** Representative growth curve of hfNSCs showing the increase in cell number over time (cells ×10^4^, line plot with blue markers). Growth curves were generated for each donor-derived line; one representative example is shown. **(B)** Representative confocal images showing the expression of canonical neural stem cell/progenitor markers Sox-2, Nestin, CD15 and Musashi-1 in hfNSCs. For each marker, left panels show merged images with F-actin (phalloidin) in green and marker staining in blue; right panels show the corresponding marker channel (blue) alone. Images were acquired from hfNSCs derived from Donor 1 and are representative of the staining pattern observed across all three donor-derived lines. Scale bar: 20 µm (applies to all panels).

hfNSC cultures derived from three independent donors were analyzed throughout the study. To document their proliferative capacity *in vitro*, growth curves were generated for each donor line; a representative example is shown in Figure 1A, illustrating a progressive increase in cell number over time. Confocal microscopy revealed expression of the canonical stemness markers Sox-2, Nestin, CD15, and Musashi-1 across hfNSC cultures (Figure 1B). In merged images, marker immunoreactivity was visualized in relation to the F-actin cytoskeleton, used as a structural reference to outline cell morphology and to contextualize marker subcellular localization: Sox-2 was predominantly nuclear, Nestin and Musashi-1 showed mainly perinuclear and cytosolic staining, and CD15 showed a membrane-associated localization. Similar expression patterns were observed across all three donor-derived hfNSC lines (data not shown), supporting a neural stem/progenitor phenotype.

### 3.2 Resting membrane potential and excitability profile

**FIGURE 2:**
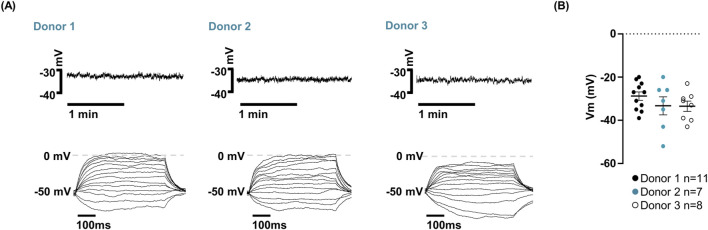
Analysis of the resting membrane potential in hfNSCs. **(A)** Representative whole-cell current-clamp recordings from three distinct hfNSC donors (Donor 1, Donor 2, and Donor 3). Top: 2-min gap-free recordings obtained in current-clamp mode without current injection. Bottom: responses to depolarizing current steps applied in 5 pA increments (step duration 500 m). No spike firing was evoked in response to current injection. **(B)** Scatter plots of resting membrane potential values from three independent donors (Donor 1: black dots, n = 11; Donor 2: cyan dots, n = 7; Donor 3: open dots, n = 8). No significant differences in resting membrane potential were detected across donors (Donor 1: −28.8 ± 1.93 mV; Donor 2: −33.29 ± 4.18 mV; Donor 3: −33.50 ± 2.36 mV). Data are expressed as mean ± SEM and analyzed using one-way ANOVA for multiple comparison (Donor 1 vs. Donor 2: p-value = 0.4841; Donor 1 vs. Donor 3: p-value = 0.4246; Donor 2 vs. Donor 3: p-value = 0.9985).

Whole-cell patch-clamp analysis revealed that hfNSCs from all three donors showed a highly conserved bioelectrical phenotype. The Vm, measured in current-clamp mode without current injection, was consistently depolarized, averaging −31.46 ± 1.56 mV with no significant differences across donors (Donor 1: n = 11; Donor 2: n = 7; Donor 3: n = 8, Figures 2A,B). No spontaneous action potentials were observed in gap-free recordings, and depolarizing current steps (5 pA increments) failed to evoke action potentials, confirming the non-excitable state typical of undifferentiated cells (Figure 2A).

### 3.3 Voltage-gated outward currents: Activation properties

**FIGURE 3:**
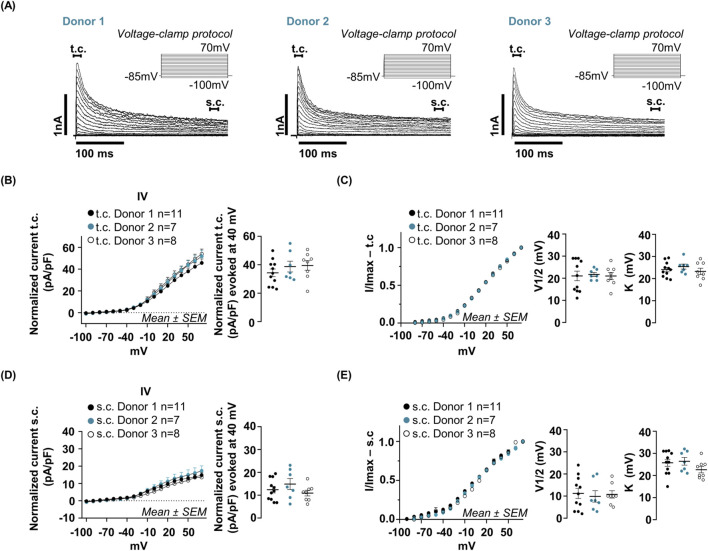
Characterization of voltage-gated currents in hfNSCs. **(A)** Representative current profiles recorded from three distinct hfNSC donors (Donor 1, Donor 2 and Donor 3) using the whole-cell voltage clamp configuration. Quantifications in panels B–E were obtained from the same cells: Donor 1 (n = 11), Donor 2 (n = 7), Donor 3 (n = 8). Voltage-dependent currents were elicited by 300 m depolarizing steps from −100 mV to +70 mV (in 10 mV increments, holding potential at −85 mV), normalized to membrane capacitance (pA/pF), and plotted as current-voltage (I–V) relationships. All donors showed a shared current profile characterized by a rapid activating outward transient component that decays within a few milliseconds to a sustained component persisting throughout the step No inward currents were detected. Based on their kinetics, two components were analyzed separately: a transient current (t.c.), defined by rapid activation (peak around 10 m after step onset), large amplitude, and fast inactivation; and a sustained current (s.c.), remaining clearly detectable at the end of the 300 m trace. Peak values of the t. c. were measured between 0–20 m **(B,C)**, whereas s. c. was measured between 280–300 m **(D,E)**. **(B)** I–V relationship for the t. c. (Donor 1: black dots; Donor 2: cyan dots; Donor 3: open dots). The t. c. activated at around −40 mV and increased with further depolarization. The t. c. amplitude at +40 mV was comparable across donors (mean ± SEM): Donor 1: 34.35 ± 2.6 pA; Donor 2: 38.70 ± 3.7 pA; Donor 3: 39.50 ± 3.3 pA. One-way ANOVA: statistical analysis confirmed there was no significant difference in current t. c. amplitude between donors (Donor 1 vs. Donor 2: p-value = 0.61; Donor 1 vs. Donor 3: p-value = 0.4780; Donor 2 vs. Donor 3: p-value = 0.9855). **(C)** Activation kinetics of the t. c. Normalized t. c. (I/I_max_) was fitted with a Boltzmann function to derive the half-maximal activation voltage (V_1_/_2_) and the slope factor (K). Symbols as in **(B)**. The V_1_/_2_ and K values exhibited robust consistency across the three analyzed donors. V_1_/_2_ (mean ± SEM): 21.05 ± 2.15 mV for Donor 1, 21.71 ± 0.89 mV Donor 2 and 21.00 ± 1.61 mV Donor 3; One way ANOVA comparison of V_1_/_2_: Donor 1 vs. Donor 2: p-value = 0.96; Donor 1 vs. Donor 3: p-value = 0.99; Donor 2 vs. Donor 3: p-value = 0.96. K (mean ± SEM): 24.09 ± 1.01 mV for Donor 1, 25.43 ± 1.11 mV Donor 2 and 23.25 ± 1.44 mV Donor 3; One way ANOVA comparison of (K) Donor 1 vs. Donor 2: p-value = 0.71; Donor 1 vs. Donor 3: p-value = 0.86; Donor 2 vs. Donor 3: p-value = 0.46). **(D)** I–V relationship for the s. c. Symbols as in **(B)**. The s. c. activated at approximately −40 mV and increased with depolarization. The s. c. amplitude at +40 mV was comparable across donors (mean ± SEM): Donor 1: 12.41 ± 1.41 pA; Donor 2: 14.90 ± 2.42 pA; Donor 3: 10.92 ± 1.24 pA. One-way ANOVA: statistical analysis confirmed there was no significant difference in current s. c. amplitude between donors (Donor 1 vs. Donor 2 p-value = 0.55; Donor 1 vs. Donor 3: p-value = 0.79; Donor 2 vs. Donor 3: p-value = 0.55). **(E)** Activation kinetics of the s. c. Normalized s. c. (I\I_max_) was fitted to a Boltzmann function (symbols as in B) to derive V_1/2_ and K. The V_1/2_ values were highly consistent across donors (mean ± SEM): Donor 1 : 11.21 ± 2.27 mV; Donor 2: 9.85 ± 2.59 mV; Donor 3 = 10.75 ± 1.65 mV. One-way ANOVA for multiple comparison confirmed no significant differences in the V_1/2_ between donors (Donor 1 vs. Donor 2: p-value = 0.96; Donor 1 vs. Donor 3: p-value = 0.99; Donor 2 vs. Donor 3: p-value = 0.96). Similarly, K values were homogeneous (mean ± SEM): Donor 1: 25.74 ± 1.58 mV; Donor 2: 26.43 ± 1.73 mV; Donor 3: 22.63 ± 1.43 mV. One way ANOVA comparison of K: Donor 1 vs. Donor 2: p-value = 0.95; Donor 1 vs. Donor 3: p-value = 0.35; Donor 2 vs. Donor 3: p-value = 0.28.

Whole-cell voltage-clamp recordings revealed a composite voltage-gated outward current consisting of two kinetically distinct components: a transient, rapidly inactivating current (t.c.) and a sustained, non-inactivating component (s.c.) (Figure 3A). Both components were present in all donors and showed highly reproducible amplitudes and kinetics across preparations. The t. c. was activated at approximately −40 mV and reached its maximum amplitude at +70 mV, the highest potential tested, displaying rapid activation followed by complete inactivation within tens of milliseconds. Current–voltage relationships showed overlapping activation profiles across donors, with no significant differences in current amplitude, and an averaged peak amplitude of 37.52 ± 3.43 pA at +40 mV (Figure 3B). Boltzmann fits confirmed consistency between donors, with an average V_1_/_2_ of 21.25 ± 1.56 mV and an average K of 24.22 ± 1.20 mV (Figure 3C). The s. c. activated at a voltage threshold comparable to the t. c., beginning at approximately −40 mV. Its amplitude increased progressively with membrane depolarization, reaching maximal values at the highest potential tested (+70 mV). In contrast to the transient component, s. c. remained stable throughout the entire 500 m depolarizing step, showing no appreciable inactivation. At corresponding voltages, the peak amplitude of s. c. was consistently lower than that of t. c., being on average approximately threefold smaller. The mean s. c. amplitude measured at +40 mV was 12.7 ± 1.6 pA, with no significant differences observed among donors (Figure 3D). Boltzmann analysis indicated consistency of s. c. activation properties, with an average V_1_/_2_ of 10.6 ± 1.9 mV and an average K of 24.9 ± 1.6 mV (Figure 3E). All quantitative parameters reported in Figures 3B–E were obtained from the same set of cells: Donor 1 (n = 11), Donor 2 (n = 7), Donor 3 (n = 8).

### 3.4 Distinct inactivation kinetics define two biophysically separable outward currents

**FIGURE 4:**
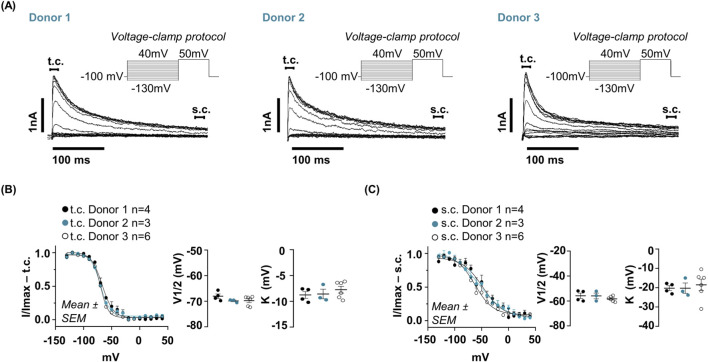
Steady-state inactivation of voltage-gated currents in hfNSCs. **(A)** Representative whole-cell voltage-clamp recordings from hfNSCs derived from three independent donors showing comparable steady-state inactivation kinetics. Inactivation was assessed by applying 500 m conditioning pre-pulses ranging from −100 mV to +40 mV (10 mV increments), followed by a 300 m test pulse to +50 mV. **(B)** Peak amplitudes of the t. c. were measured between 0 and 20 m while **(C)** the s. c. was analyzed between 280 and 300 m. Quantifications in panels B–C were obtained from the same cells: Donor 1 (n = 4, black dots), Donor 2 (n = 3, cyan dots), Donor 3 (n = 6, open dots). Current amplitudes were normalized to their respective maximal values and fitted with a Boltzmann function to determine V_1_/_2_ and K. The t. c. showed complete inactivation around −50 mV, whereas the s. c. inactivated at more depolarized potentials, with complete inactivation occurring near 0 mV. No significant donor-dependent variations were observed in the steady-state inactivation kinetics of either current type. For the transient component, V_1_/_2_ (mean ± SEM) values were −68.01 ± 0.8789 mV in Donor 1, –69.86 ± 0.33 mV in Donor 2, and –69.76 ± 0.76 mV in Donor 3. Corresponding K values (mean ± SEM) were −8.76 ± 0.65 mV (Donor 1), −8.58 ± 0.93 mV (Donor 2), and −7.75 ± 0.65 mV (Donor 3). One-way ANOVA confirmed no significant differences across donors (V_1_/_2_: Donor 3 vs. Donor 2, p-value = 0.96; Donor 3 vs. Donor 1, p-value = 0.99; Donor 2 vs. Donor 1, p-value = 0.96; K: Donor 3 vs. Donor 2, p-value = 0.64; Donor 3 vs. Donor 1, p-value = 0.59; Donor 2 vs. Donor 1, p > 0.99). For the s. c., the V_1_/_2_ (mean ± SEM) was −55.82 ± 2.12 mV (Donor 1), −55.94 ± 2.28 mV (Donor 2), and −58.26 ± 0.84 mV (Donor 3). The corresponding K values (mean ± SEM) were 22.63 ± 1.44 mV (Donor 1), 26.43 ± 1.73 mV (Donor 2), and 25.74 ± 1.59 mV (Donor 3). One-way ANOVA showed no significant inter-donor differences (V_1_/_2_: Donor 3 vs. Donor 2, p-value = 0.96; Donor 3 vs. Donor 1, p-value = 0.99; Donor 2 vs. Donor 1, p-value = 0.96; K: Donor 3 vs. Donor 2, p-value = 0.64; Donor 3 vs. Donor 1, p-value = 0.59; Donor 2 vs. Donor 1, p-value >0.99). Together, these findings demonstrate highly consistent inactivation kinetics across hfNSC lines from different donors, supporting a conserved pattern of voltage-dependent regulation of K^+^ conductances within this cell population.

Steady-state inactivation was assessed using conditioning pre-pulses ranging from −100 to +40 mV (in 10 mV increments) followed by a test pulse at +50 mV. This analysis revealed highly similar inactivation profiles across all donors (Figure 4A). The transient component inactivated completely at approximately −50 mV (Figure 4B), whereas the sustained component required more depolarized potentials, with half-inactivation occurring near 0 mV (Figure 4C). Quantitative Boltzmann fitting confirmed strong inter-donor consistency: the t. c. exhibited an average V_1/2_ of approximately −69 ± 1.5 mV with a slope (K) of roughly −8 ± 1 mV, while the s. c. showed a mean V_1/2_ of approximately −56 ± 2 mV and an average K value of 24 ± 2 mV. No statistically significant differences were detected between donors. This distinct inactivation profile between the two components provides a robust biophysical criterion for discriminating them and further reinforces the donor-independent nature of the hfNSC electrophysiological signature. All data shown in Figures 4B,C were obtained from the same set of cells: Donor 1 (n = 4), Donor 2 (n = 3), Donor 3 (n = 6).

### 3.5 Biophysical and pharmacological isolation of current components

**FIGURE 5:**
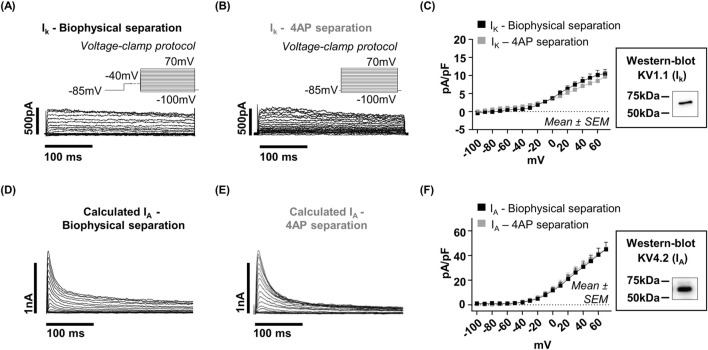
Biophysical and pharmacological separation of the two components of the composite outward current recorded in hfNSCs. **(A)** A 500 m pre-pulse to −40 mV was applied to inactivate the transient current, thereby isolating the s. c. (measured at +40 mV, mean ± SEM: 8.46 ± 1.04 pA/pF, n = 9). **(B)** Pharmacological inhibition using 4-aminopyridine (4-AP) selectively blocked the t. c., allowing isolation of the s. c. (at +40 mV, mean ± SEM: 7.09 ± 0.87 pA/pF, n = 9). **(D,E)** The t. c. was calculated by digitally subtracting the s. c. from the total composite current in each condition (see Figure 3A for total composite current representative traces), yielding values (mean ± SEM) of: 30.79 ± 4.08. pA/pF following biophysical separation, and 32.25 ± 3.97 pA/pF after 4-AP inhibition (both n = 9). **(C,F)** I–V plots of the isolated components are shown, along with Western blot analysis. The t. c., which is inhibited by both the −40 mV pre-pulse (black square) and 4-AP (grey square), is consistent with an A-type K^+^ current (I_A_). Conversely, the non-inactivating, 4-AP-insensitive sustained component is consistent with a delayed rectifier K^+^ current (I_K_). Western blot analysis confirmed the expression of two channel subtypes, Kv4.1 and Kv1.1, consistent with the I_A_ and the I_K_ currents identified electrophysiologically. No statistically significant differences were found between the two separation methods (two-way ANOVA), which supports the validity and reliability of both approaches. Each experimental condition represents a homogeneous sample (n = 9) derived from the three independents donors.

Based on the distinct activation and inactivation kinetics observed for the two outward current components, we hypothesized that they correspond to two functionally different classes of K^+^ currents: a fast, transient A-type current (I_A_) and a sustained delayed-rectifier current (I_K_). To test this hypothesis and to discriminate between the two components, we used a combined biophysical and pharmacological approach. All experiments and quantifications shown in Figure 5 were performed on 9 cells, 3 cells per donor. Biophysically, a pre-pulse of −40 mV selectively inactivated the fast transient component, thereby isolating the sustained current (Figure 5A). Pharmacologically, 4-aminopyridine (4-AP), a selective I_A_ blocker, was applied to separate the two components (Figure 5B). Both methods produced similar results (Figures 5C–F), confirming the presence of a transient, 4-AP-sensitive current and a sustained, 4-AP-resistant component. Based on their distinct kinetic profiles and pharmacological responses, we unambiguously identified the transient component as I_A_ and the sustained component as I_K_.

**FIGURE 6:**
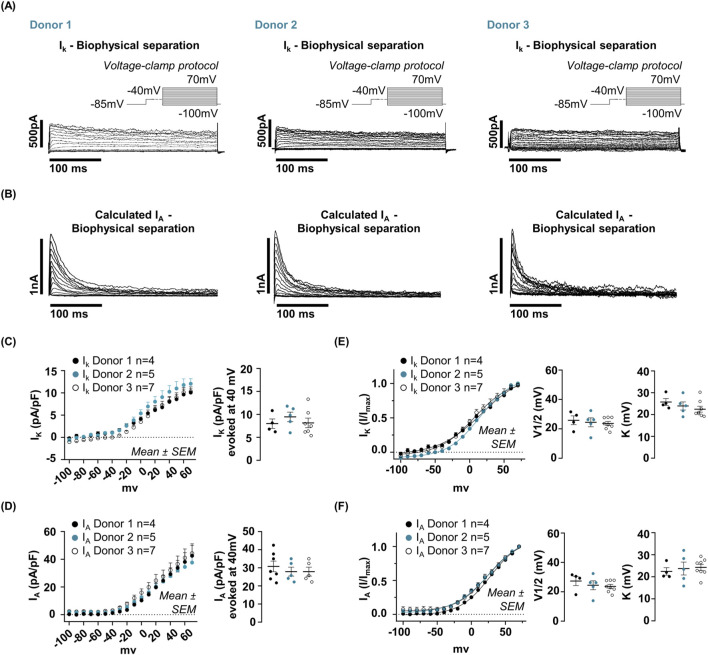
Comparative analysis of isolated K^+^ current components in hfNSCs derived from different donors. **(A)** Representative whole-cell voltage-clamp recordings obtained from three independent donors (Donor 1, Donor 2, Donor 3), demonstrating the biophysical isolation of the sustained delayed-rectifier K^+^ current (I_K_). A 500 m pre-pulse of −40 mV was used to selectively inactivate the transient A-type current (I_A_). For each condition, the corresponding I_A_ trace obtained by digital subtraction is also displayed. **(B)** shows representative traces of the digitally reconstructed I_A_ component. Quantifications in panels C–F were obtained from the same cells: Donor 1 (n = 4, black dots), Donor 2 (n = 5, cyan dots), Donor 3 (n = 7, open dots). **(C)** Current density (pA/pF) of the isolated I_K_ component measured at +40 mV demonstrated high consistency across groups of hfNSCs derived from different donors (Donor 1: 7.99 ± 1.03; Donor 2: 9.45 ± 1.04; Donor 3: 8.14 ± 1.06). **(D)** The current density (pA/pF) of the isolated I_A_ component, measured at +40 mV, was homogeneous among donors (Donor 1: 30.76 ± 2.92 pA/pF; Donor 2: 27.90 ± 2.48 pA/pF; Donor 3: 28.12 ± 2.51 pA/pF). **(E)** Normalized I_K_ currents (I/I_max_) plotted as activation curves revealed overlapping voltage dependencies across all donor-derived lines, with comparable activation parameters. The mean ± SEM for V_1/2_ is given below: For Donor 1, 27.34 ± 3.14 mV; for Donor 2, 24.46 ± 3.01 mV; and for Donor 3, 23.66 ± 1.36 mV. The mean values for K were 25.74 ± 1.62 for Donor 1, 23.94 ± 1.84 for Donor 2, and 22.46 ± 1.35 for Donor 3. **(F)** Furthermore, analysis of normalized I_A_ activation curves demonstrated a high degree of consistency across various donor groups. V_1/2_ (mean ± SEM) are as follows: 25.84 ± 2.99 mV for Donors 1, 24.46 ± 3.01 mV for Donor 2 and 23.66 ± 1.36 mV for Donor 3. K (mean ± SEM): 22.45 ± 1.70 for Donor 1, 23.75 ± 2.93 for Donor 2, and 24.28 ± 1.36 for Donor 3.

Molecular validation by Western blot demonstrated the expression of Kv4.2, functionally associated with I_A_, and Kv1.1, linked to I_K_, corroborating the electrophysiological classification. As the −40 mV pre-pulse protocol reliably inactivated the I_A_ across all donors, this approach was adopted for subsequent analyses to separate the two current components (Figure 6). Quantitative analysis of the isolated current components revealed homogeneity across donors. All electrophysiological quantifications shown in Figures 6C–F were obtained from the same set of cells: Donor 1 (n = 4), Donor 2 (n = 5), Donor 3 (n = 7). At +40 mV, the average current density for I_K_ was 8.53 ± 1.04 pA/pF, and the average density for I_A_ was 28.93 ± 2.64 pA/pF (Figures 6C,D). Similarly, both components exhibited nearly identical activation parameters across all donors: I_K_ showed an average V_1/2_ of approximately 25 ± 2 mV with an average slope factor (K) of 24 ± 2 mV, and I_A_ showed an average V_1/2_ of approximately 25 ± 2 mV and a mean slope factor (K) of 23 ± 1 mV (Figures 6E,F). No significant differences were observed in any of these analyzed parameters across hfNSCs from different donors. Together, these data demonstrate that hfNSCs from distinct fetal sources possess a conserved, donor-independent electrophysiological signature defined by the stable coexistence of two K^+^ currents (I_A_ and I_K_), alongside a depolarized Vm and a non-excitable state. This remarkable uniformity across independent donors provides a robust bioelectrical signature for SVZ-derived neural progenitors.

### 3.6 Electrophysiological dynamics during differentiation

**FIGURE 7:**
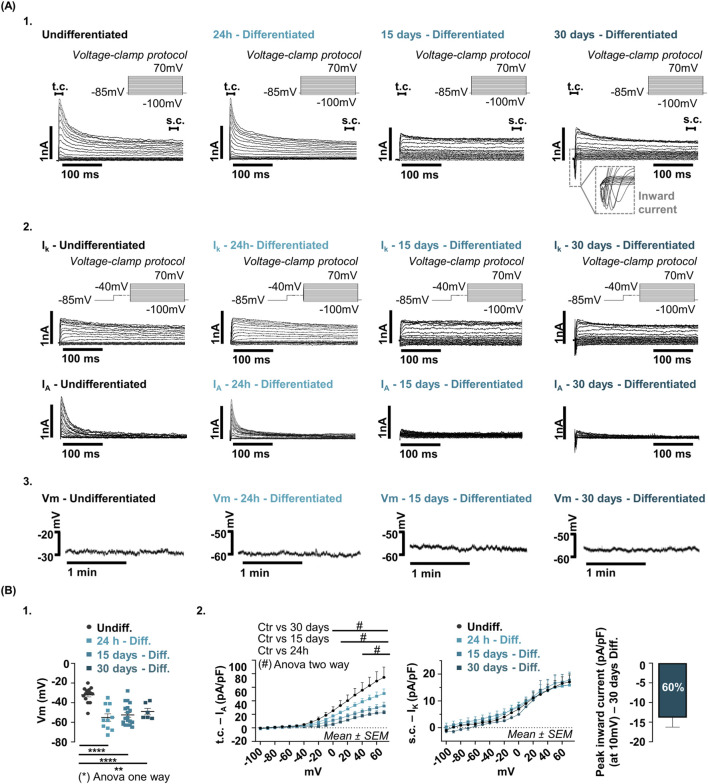
Biophysical profile of hfNSCs during differentiation. **(A)** Representative whole-cell patch-clamp recordings from undifferentiated (Undiff.) hfNSCs and from differentiated (Diff.) cells at 24 h, 15 days, and 30 days after-induction. (A1) Voltage-clamp recordings showing evoked currents in response to 300 m depolarizing steps from −100 mV to +70 mV (10 mV increments) from a holding potential of −85 mV. (A2) Biophysically separation of I_K_ and I_A_ current components at the indicated time points. I_K_ was isolated using a 500 m pre-pulse to −40 mV and recorded during 300 m depolarizing steps from −100 mV to +70 mV (10 mV increments); I_A_ was obtained by digital subtraction. (A3) Vm recorded for 2 min in current-clamp mode without current injection. Representative traces illustrate differentiation-associated changes in bioelectrical properties over time. **(B)** Quantitative analysis of electrophysiological parameters across differentiation stages. (B1) Analysis of Vm during differentiation. Vm values were measured in undifferentiated hfNSCs (n = 14, black dots) and in differentiated cells as 24 h (n = 11, light blue squares), 15 days (n = 19, blue squares) and 30 days post-induction (n = 6, dark blue squares). Vm displayed a significant and sustained hyperpolarization beginning at 24 h after induction. Vm values (mean ± SEM) were: Undiff. = −32.00 ± 2.08 mV; 24 h - Diff. = −52.47 ± 2.47 mV; 15 days–Diff. = −49.00 ± 3.29 mV; 30 days–Diff. = −49.00 ± 3.29 mV. Statistical analysis was performed using a one-way ANOVA test, comparing each time point with the undifferentiated condition: Undiff. vs. 24 h - Diff., ****p-value <0.0001; Undiff. vs. 15 days - Diff., **** p-value <0.0001; Undiff. vs. 30 days–Diff., **p-value = 0.003. (B2) I–V relationships for I_A_ and I_K,_ and peak inward current amplitude recorded at +10 mV at 30 days after induction. Differentiation-associated current remodeling was analyzed in undifferentiated hfNSCs (n = 10 cells, black dots) and in differentiated cells at 24 h (n = 10 cells, light blue squares), 15 days (n = 9 cells, blue squares), and 30 days after induction (n = 5 cells, dark blue squares). The transient outward component (t.c., I_A_) decreased progressively and significantly during differentiation. Two-way ANOVA revealed significant differences (indicated by # in the graph). At 24 h of differentiation, the reduction became significant from +40 mV onward (p-value = 0.02 to p-value <0.0001). At day15, significance was first detected at 0 mV (p-value = 0.03) and became highly significant between +10 and +70 mV (p-value <0.0001 at +40 mV). A similar pattern was observed at 30 days of differentiation, with the significant reduction from 0 mV (p-value = 0.03) to +70 mV (p-value <0.0001). In contrast, the sustained component (s.c., I_K_) remained unchanged throughout differentiation and became the predominant outward conductance by day 15. Two-way ANOVA confirmed no significant differences in I_K_ amplitude across time points (p-value >0.05 for all tested voltages). By 30 days post-differentiation, inward currents were detected in approximately 60% of recorded cells (3 out of 5), with an average amplitude of −13.86 ± 2.42 pA/pF.

To evaluate how differentiation shapes the electrophysiological profile of hfNSCs, recordings were performed 24 h, 15 days and 30 days after induction (Figure 7A). Upon differentiation induction, resting Vm hyperpolarized within 24 h, shifting from −32.00 ± 2.08 mV in undifferentiated cells (n = 14) to −55.18 ± 3.75 mV (n = 11). This hyperpolarized state remained stable at 15 and 30 days (−52.47 ± 2.47 mV, n = 19 and −49.00 ± 3.29 mV, n = 6, respectively), indicating that Vm undergoes an early and sustained adjustment during differentiation progression (Figures 7B–1). The timing of this shift suggests that Vm behaves as an early functional marker of the onset of differentiation. Importantly, throughout the entire differentiation time course, depolarizing current injections failed to elicit self-sustained spike firing, indicating that hfNSCs remain non-excitable under our recording conditions up to day 30 (Supplementary Figure S1).

Differentiation-associated current remodeling was assessed in undifferentiated hfNSCs (n = 10 cells) and at 24 h (n = 10 cells), 15 days (n = 9 cells), and 30 days after induction (n = 5 cells). Analysis of voltage-gated outward currents revealed a progressive remodeling of K+ conductances. I_A_ decreased significantly over time, with reduction evident at 24 h and markedly reduced at later stages (Figures 7B–2). In contrast, I_K_ remained essentially unchanged and became the predominant outward conductance by day 15. This pattern indicates that early differentiation involves selective downregulation of I_A_ while preserving I_K_. At later stages, inward voltage-dependent currents appeared by day 30 in approximately 60% of recorded cells (3 out of 5), with a mean amplitude of −13.86 ± 2.42 pA at +10 mV (Figures 7B–2). These inward currents, likely mediated by voltage-gated Na^+^ or Ca^2+^ channels, suggest the emergence of a neuro-glial electrophysiological phenotype in a fraction of differentiating hfNSCs.

Overall, differentiation in hfNSCs proceeds through a temporally ordered sequence of bioelectrical changes: early Vm hyperpolarization, progressive reduction of I_A_, and late acquisition of inward currents. This coordinated trajectory is consistent with an electrophysiological transition from a progenitor-like to a differentiating state and positions bioelectrical remodeling as a functional signature of differentiation-associated progression that can be used alongside molecular markers when available.

## 4 Discussion

This study provides the first comprehensive electrophysiological characterization of hfNSCs, derived from the SVZ at a defined gestational stage, and identifies a conserved bioelectrical profile shared across independent donor-derived cell lines. Although hfNSCs are well characterized in terms of molecular identity and lineage commitment potential, their electrophysiological features have received limited attention. Here, we show that SVZ-derived hfNSCs, corresponding to the same cell populations previously demonstrated to possess self-renewal capacity and neuroprotective activity (Capobianco et al., 2024), consistently express canonical stemness markers including Nestin, Sox-2, Musashi-1, and CD15. This well-established molecular identity is paralleled by a highly reproducible and donor-independent bioelectrical phenotype. The convergence of marker expression and a stereotyped bioelectrical signature support the view that Vm and voltage-gated conductances are integral components of progenitor-state identity rather than a secondary by-product of culture conditions. In addition, hfNSCs displayed sustained proliferative capacity *in vitro*.

By systematically comparing hfNSCs from 3 independent donor-derived preparations, our findings define a preserved electrical signature of the progenitor state. hfNSCs display a highly consistent bioelectrical phenotype characterized by a stably depolarized Vm, a non-excitable profile, and a stereotyped combination of K+ currents, in which a large transient A-type component is balanced by a sustained delayed-rectifier component. Notably, this electrical configuration partly resembles that reported in human fetal midbrain progenitors (Schaarschmidt et al., 2009), despite their distinct developmental origins and neurogenic functions. However, in the midbrain model, the increase in I_K_ during differentiation shifts the I_A_/I_K_ balance in a way that differs significantly from the SVZ-derived hfNSC differentiation profile. Midbrain-derived NSCs, primarily used as models for dopaminergic neurogenesis, represent a more restricted population than SVZ-derived hfNSCs, which possess broader neuro-glial differentiation capacity (Andreotti et al., 2019). The fact that both populations share a similar bioelectrical signature despite these functional differences suggests that this electrical configuration may represent a conserved property of neural progenitor identity. Consistent with a conserved bioelectrical configuration, neonatal rat SVZ neural stem cells also display a depolarized resting potential, non-excitability, and I_A_/I_K_ current profile (Stewart et al., 1999). This cross-regional and cross-species conservation suggests that the signature reflects a fundamental biophysical property of neural progenitors. Together, these findings position bioelectrical profiling as a robust, quantifiable functional parameter for hfNSC identity, providing an objective complement to molecular marker-based approaches, with potential utility for establishing standardized electrophysiological benchmarks in GMP-grade hfNSC production (Gelati et al., 2022).

Notably, the stability of this electrophysiological signature distinguishes hfNSCs from other stem cell models in which functional heterogeneity is a major limitation. For example, adult mesenchymal stem cells (MSCs) show donor-to-donor variability driven by age, tissue source, and progressive functional decline during long-term culture (Česnik and Švajger, 2024). Similarly, induced pluripotent stem cells (iPSCs) can retain residual epigenetic memory from the donor tissue, leading to heterogeneous electrophysiological profiles that affect differentiation reproducibility (Nath et al., 2023), post-graft integration and *in vivo* performance (Nori et al., 2011; Grealish et al., 2014). In this scenario, the functional stability observed in SVZ-derived hfNSCs may offer a significant advantage for quality control and standardization in regenerative strategies (Karanu et al., 2020).

A key observation of this study is the speed of Vm hyperpolarization, which occurs within 24 h of differentiation onset. This functional shift precedes the downregulation of canonical stemness markers such as Sox-2 and Nestin, whose expression typically persists for 2–5 days in human and rodent neural progenitor before declining (Conti et al., 2005; Pollard et al., 2006; Elkabetz et al., 2008). Consistently, early Vm changes have been reported in cerebellar granule progenitors and embryonic NSCs, where hyperpolarization precedes the upregulation of neuronal markers such as βIII-tubulin and NeuN by 2–3 days (Sundelacruz et al., 2009; Vitali et al., 2018). Together, these temporal dynamics suggest that bioelectric remodeling is an early functional event that anticipates transcriptional commitment, positioning Vm as a potential early indicator of lineage progression. While our data do not establish causality, they align with evidence from other progenitor systems in which Vm modulation actively influences fate specification, including cerebellar and embryonic hNSCs (Sundelacruz et al., 2009; Vitali et al., 2018; Levin, 2014). Comparable early Vm shifts have also been observed in myogenic and epithelial models, where Kir2.1-dependent hyperpolarization precedes the expression of transcriptional regulators such as MyoD and Myogenin (Konig et al., 2004; Hinard et al., 2008). Similar voltage-dependent transitions have been reported in embryonic stem cells and during epithelial-to-mesenchymal transitions, where membrane polarization modulates downstream Ca^2+^/CaMK and MAPK signalling to guide lineage commitment (Sundelacruz et al., 2009; Levin, 2014; Chen et al., 2014). We therefore hypothesize that the temporal precedence of Vm hyperpolarization might function as a bioelectric cue that can influence downstream transcriptional and proteomic remodeling during lineage progression. Testing this causality will require integrated, time-resolved measurements combining electrophysiology with transcriptomics and proteomics on matched time points, ideally coupled to Vm perturbation paradigms to determine whether enforced hyperpolarization/depolarization reshapes the molecular trajectory.

We therefore propose a three-stage model of electrical maturation in hfNSCs in which distinct mechanisms act sequentially. During the first 24 h, rapid Vm hyperpolarization occurs in the absence of any detectable changes in I_K_ or I_A_ at physiologically relevant voltages. This suggests that early bioelectric remodelling involves mechanisms that are independent of voltage-gated K^+^ channels, such as Na^+^/K^+^-ATPase activity or modulation of leak channels (K2P, HCN). This temporal dissociation mirrors observations in other progenitor systems where Vm shifts precede measurable ion channel remodeling (Sundelacruz et al., 2009; Levin, 2014; McLaughlin and Levin, 2018). While our data do not identify the specific mechanisms underlying the Vm shift, plausible candidates based on related progenitor systems include increased Na^+^/K^+^-ATPase activity or surface density (Blackiston et al., 2009), upregulation of background K2P channels (Ter and And, 2010), and altered intracellular Cl-homeostasis via NKCC1/KCC2 transporters or gap-junction coupling (Yamada et al., 2004; Ben-Ari, 2014; Talaverón et al., 2015). Taken together, these findings support a model in which early bioelectric remodeling reflects rapid adjustments in ionic transport and passive conductances, preceding the voltage-gated channel reorganization.

Once the initial hyperpolarization has occurred, the remodeling of voltage-gated K^+^ conductances consolidates this transition. In undifferentiated hfNSCs, I_A_ (Kv4.2) and I_K_ (Kv1.1) maintain Vm within the depolarized range (−30 to −50 mV) typical of progenitor-like states (Schaarschmidt et al., 2009; Morokuma et al., 2008). The selective reduction of I_A_ during differentiation removes this stabilizing influence, allowing Vm to shift toward more negative values. This I_A_ downregulation is consistent with previous studies linking Kv4-family suppression to neuronal differentiation (Schaarschmidt et al., 2009), although it remains unclear whether this reflects reduced Kv4.2 expression, altered trafficking, or regulatory phosphorylation events (Covarrubias et al., 2008). Notably, at the depolarized resting potentials typical of hfNSCs (around −30 mV), I_A_ channels are largely inactivated and contribute minimally to the steady-state conductance; instead, I_A_ functions as a fast electrical stabilizer, generating brief outward currents that damp voltage fluctuations (Fröhlich et al., 2008). As I_A_ diminishes, this stabilizing mechanism is lost, allowing the membrane to adopt the hyperpolarized profile associated with later stages of differentiation. Variations in the I_A_/I_K_ ratio thus track the progressive transition from a progenitor-like to a more differentiated state (Schaarschmidt et al., 2009). With Vm stabilization complete, cells transition to acquire more mature electrophysiological features. Consistently, fast-inactivating voltage-dependent inward currents emerge in approximately 60% of cells by day 30, suggesting progression toward more differentiated neuro-glial electrophysiological states (Biella et al., 2007; Song et al., 2013). Notably, despite the appearance of inward currents, depolarizing current injections failed to elicit spike firing on day 30, indicating that cells remain non-excitable under our recording conditions. In the remaining cells, inward currents were not detected at this time point, consistent with heterogeneity in differentiation trajectories and/or maturation stage. However, the molecular identity of the voltage-dependent inward currents was not determined here (e.g., by pharmacology), and lineage assignment will require correlation with appropriate neuronal and glial markers.

The depolarized Vm observed in undifferentiated hfNSCs (about −30 mV) aligns with values reported for other neural progenitors, where such potentials are associated with proliferation and self-renewal (Sundelacruz et al., 2009). While we document proliferative capacity at the population level, we did not assess cell-cycle stage in the recorded cells; therefore, links between Vm/ion currents and cell-cycle regulation should be considered hypothesis-driven. This bioelectrical configuration likely maintains the undifferentiated state by functionally silencing Ca^2+^-dependent signalling pathways. In line with this, T-type (Cav3.1/3.2) and L-type (Cav1.2/1.3) Ca^2+^ channels are weakly expressed and largely inactivated at these depolarized potentials (D’Ascenzo et al., 2006; Guo et al., 2010), thereby preventing activation of CaMKII, CREB, and MAPK/ERK cascades that drive differentiation (Hinard et al., 2008; Berridge, 1995). The absence of detectable voltage-gated Ca^2+^ currents in our hfNSCs recordings further supports this non-excitable, progenitor-like electrophysiological profile. A depolarized Vm may support the proliferative and undifferentiated state of hfNSCs through multiple mechanisms. First, Vm modulates K-Ras GTPase activity, a key G1-to-S phase regulator, by altering electrostatic interactions with plasma membrane phospholipids. Depolarization reduces K-Ras tethering, activating ERK/MAPK and mTOR signalling cascades (Zhou et al., 2015; Sempou et al., 2022; Kadir et al., 2018) that drive Cyclin D expression and mitotic entry (Vadlakonda et al., 2013; Benary et al., 2020). This K-Ras/ERK axis is particularly relevant to neural progenitor self-renewal (Bender et al., 2015). Second, depolarization stabilizes β-catenin, reinforcing Wnt-dependent self-renewal programs (Rapetti-Mauss et al., 2020). Third, bioelectric state influences metabolic activity and cytosolic/endosomal pH via Na^+^/H^+^ exchangers, regulating growth factor signalling duration (Sundelacruz et al., 2009; Rapetti-Mauss et al., 2020). These mechanisms, while not yet demonstrated in hfNSCs, provide a plausible framework for how depolarized Vm may actively maintain progenitor identity.

### 4.1 Limitations

While our findings demonstrate reproducibility across independent hfNSC lines, all three donors were isolated from SVZ tissue collected between gestational weeks 15–16 and had comparable clinical backgrounds. The donor-independence reported here should be interpreted as reproducibility within this restricted developmental window and future work will be required to test whether the same bioelectrical signature extends to hfNSCs from different gestational ages. In addition, although we propose that hfNSCs offer advantages over MSCs and iPSCs based on published data, direct head-to-head functional comparisons were beyond the scope of this study. While we propose a three-stage model of electrical maturation, the specific ionic mechanisms underlying Vm hyperpolarization remain hypothetical and will require direct experimental validation. Moreover, differentiation-stage interpretation is primarily based on electrophysiological readouts; pharmacological identification of inward currents and parallel validation with neuronal/glial markers were not performed and will be required to assign lineage and staging at later time points. Finally, while providing high-resolution data, the single-cell nature of electrophysiological analysis represents a bottleneck for large-scale applications. At present, it should be considered a specialized, high-level validation tool rather than a high-throughput screening method for routine quality assurance.

### 4.2 Conclusions

This study provides the first comprehensive electrophysiological characterization of hfNSCs derived from the human SVZ. The electrophysiological profile of progenitor-state SVZ-derived hfNSCs, defined by a depolarized Vm and a characteristic I_A_/I_K_ K^+^ current signature, is highly conserved across independent donors. The coordinated hyperpolarization of Vm and the selective remodeling of I_A_ emerge as early functional markers that track differentiation associated progression. Together, these findings establish bioelectric profiling as a robust, quantifiable framework for defining functional hallmarks of hfNSC identity and early differentiation within the 15–16-week gestational window examined here, providing objective parameters for standardizing and quality-controlling hfNSC-based therapeutic applications.

## Data Availability

The original contributions presented in the study are included in the article/Supplementary Material; further inquiries can be directed to the corresponding authors.

## References

[B1] Andreotti J. P. Silva W. N. Costa A. C. Picoli C. C. Bitencourt F. C. O. Coimbra-Campos L. M. C. (2019). Neural stem cell niche heterogeneity. Semin. Cell Dev. Biol. 95, 42–53. 10.1016/j.semcdb.2019.01.005 30639325 PMC6710163

[B2] Ben-Ari Y. (2014). The GABA excitatory/inhibitory developmental sequence: a personal journey. Neuroscience 279, 187–219. 10.1016/j.neuroscience.2014.08.001 25168736

[B3] Benary M. Bohn S. Lüthen M. Nolis I. K. Blüthgen N. Loewer A. (2020). Disentangling pro-mitotic signaling during cell cycle progression using time-resolved single-cell imaging. Cell Rep. 31 (2), 107514. 10.1016/j.celrep.2020.03.078 32294432

[B4] Bender R. H. F. Haigis K. M. Gutmann D. H. (2015). Activated k-ras, but not h-ras or N-ras, regulates brain neural stem cell proliferation in a raf/rb-dependent manner. Stem Cells 33 (6), 1998–2010. 10.1002/stem.1990 25788415 PMC4889217

[B5] Berridge M. J. (1995). Calcium signalling and cell proliferation. Bioessays 17 (6), 491–500. 10.1002/bies.950170605 7575490

[B6] Biella G. Di Febo F. Goffredo D. Moiana A. Taglietti V. Conti L. (2007). Differentiating embryonic stem-derived neural stem cells show a maturation-dependent pattern of voltage-gated sodium current expression and graded action potentials. Neuroscience 149 (1), 38–52. 10.1016/j.neuroscience.2007.07.021 17870247

[B7] Blackiston D. J. McLaughlin K. A. Levin M. (2009). Bioelectric controls of cell proliferation: ion channels, membrane voltage and the cell cycle. Cell Cycle 8 (21), 3527–3536. 10.4161/cc.8.21.9888 19823012 PMC2862582

[B8] Capobianco D. L. De Zio R. Profico D. C. Gelati M. Simone L. D’Erchia A. M. (2024). Human neural stem cells derived from fetal human brain communicate with each other and rescue ischemic neuronal cells through tunneling nanotubes. Cell Death Dis. 15 (8), 639. 10.1038/s41419-024-07005-w 39217148 PMC11365985

[B9] Česnik A. B. Švajger U. (2024). The issue of heterogeneity of MSC-based advanced therapy medicinal products-a review. Front. Cell Dev. Biol. 12, 1400347. 10.3389/fcell.2024.1400347 39129786 PMC11310176

[B10] Chen H. Luo R. Gong S. Matta S. G. Sharp B. M. (2014). Protection genes in nucleus accumbens shell affect vulnerability to nicotine self-administration across isogenic strains of adolescent rat. PLoS One 9 (1), e86214. 10.1371/journal.pone.0086214 24465966 PMC3899218

[B11] Conti L. Pollard S. M. Gorba T. Reitano E. Toselli M. Biella G. (2005). Niche-independent symmetrical self-renewal of a mammalian tissue stem cell. PLoS Biol. 3 (9), 1594–1606. 10.1371/journal.pbio.0030283 16086633 PMC1184591

[B12] Covarrubias M. Bhattacharji A. De Santiago-Castillo J. A. Dougherty K. Kaulin Y. A. Na-Phuket T. R. (2008). The neuronal Kv4 channel complex. Neurochem. Res. 33 (8), 1558–1567. 10.1007/s11064-008-9650-8 18357523 PMC5833991

[B13] Derso T. B. Mengistu B. A. Demessie Y. Fenta M. D. Getnet K. (2025). Neural stem cells in adult neurogenesis and their therapeutic applications in neurodegenerative disorders: a concise review. Front. Mol. Med. 5, 1569717. 10.3389/fmmed.2025.1569717 40612293 PMC12222294

[B14] D’Ascenzo M. Piacentini R. Casalbore P. Budoni M. Pallini R. Azzena G. B. (2006). Role of L-type Ca2+ channels in neural stem/progenitor cell differentiation. Eur. J. Neurosci. 23 (4), 935–944. 10.1111/j.1460-9568.2006.04628.x 16519658

[B15] Elkabetz Y. Panagiotakos G. Al Shamy G. Socci N. D. Tabar V. Studer L. (2008). Human ES cell-derived neural rosettes reveal a functionally distinct early neural stem cell stage. Genes Dev. 22 (2), 152–165. 10.1101/gad.1616208 18198334 PMC2192751

[B16] Fröhlich F. Bazhenov M. Iragui-Madoz V. Sejnowski T. J. (2008). Potassium dynamics in the epileptic cortex: new insights on an old topic. Neuroscientist 14 (5), 422–433. 10.1177/1073858408317955 18997121 PMC2854295

[B17] Gelati M. Profico D. C. Ferrari D. Vescovi A. L. (2022). Culturing and expansion of “Clinical Grade” neural stem cells from the fetal human central nervous system. Methods Mol. Biol. 2389, 57–66. 10.1007/978-1-0716-1783-0_5 34558001

[B18] Grealish S. Diguet E. Kirkeby A. Mattsson B. Heuer A. Bramoulle Y. (2014). Human ESC-derived dopamine neurons show similar preclinical efficacy and potency to fetal neurons when grafted in a rat model of Parkinson’s disease. Cell Stem Cell 15 (5), 653–665. 10.1016/j.stem.2014.09.017 25517469 PMC4232736

[B19] Gritti A. Frölichsthal-Schoeller P. Galli R. Parati E. A. Cova L. Pagano S. F. (1999). Epidermal and fibroblast growth factors behave as mitogenic regulators for a single multipotent stem cell-like population from the subventricular region of the adult mouse forebrain. J. Neurosci. 19 (9), 3287–3297. 10.1523/JNEUROSCI.19-09-03287.1999 10212288 PMC6782245

[B20] Guo Z. Shi F. Zhang L. Zhang H. Yang J. Li B. (2010). Critical role of L-type voltage-dependent Ca2+ channels in neural progenitor cell proliferation induced by hypoxia. Neurosci. Lett. 478 (3), 156–160. 10.1016/j.neulet.2010.05.007 20466036

[B21] Hinard V. Belin D. Konig S. Bader C. R. Bernheim L. (2008). Initiation of human myoblast differentiation via dephosphorylation of Kir2.1 K+ channels at tyrosine 242. Development 135 (5), 859–867. 10.1242/dev.011387 18216177

[B22] Kadir L. A. Stacey M. Barrett-Jolley R. (2018). Emerging roles of the membrane potential: action beyond the action potential. Front. Physiol. 9, 1661. 10.3389/fphys.2018.01661 30519193 PMC6258788

[B23] Karanu F. Ott L. Webster D. A. Stehno-Bittel L. (2020). Improved harmonization of critical characterization assays across cell therapies. Regen. Med. 15 (5), 1661–1678. 10.2217/rme-2020-0003 32589107

[B24] Konig S. Hinard V. Arnaudeau S. Holzer N. Potter G. Bader C. R. (2004). Membrane hyperpolarization triggers myogenin and myocyte enhancer factor-2 expression during human myoblast differentiation. J. Biol. Chem. 279 (27), 28187–28196. 10.1074/jbc.M313932200 15084602

[B25] Lee W. C. Shi H. Poon Z. Nyan L. M. Kaushik T. Shivashankar G. V. (2014). Multivariate biophysical markers predictive of mesenchymal stromal cell multipotency. Proc. Natl. Acad. Sci. U. S. A. 111 (42), E4409–E4418. 10.1073/pnas.1402306111 25298531 PMC4210311

[B26] Levin M. (2014). Endogenous bioelectrical networks store non-genetic patterning information during development and regeneration. J. Physiol. 592 (11), 2295–2305. 10.1113/jphysiol.2014.271940 24882814 PMC4048089

[B27] Li Z. (2013). CD133: a stem cell biomarker and beyond. Available online at: http://www.ehoonline.org/content/2/1/17. 10.1186/2162-3619-2-17 PMC3701589 23815814

[B28] Mazzini L. Gelati M. Profico D. C. Sgaravizzi G. Projetti Pensi M. Muzi G. (2015). Human neural stem cell transplantation in ALS: initial results from a phase I trial. J. Transl. Med. 13 (1), 17. 10.1186/s12967-014-0371-2 25889343 PMC4359401

[B29] Mazzini L. Gelati M. Profico D. C. Sorarù G. Ferrari D. Copetti M. (2019). Results from phase I clinical trial with intraspinal injection of neural stem cells in amyotrophic lateral sclerosis: a long‐term outcome. Stem Cells Transl. Med. 8 (9), 887–897. 10.1002/sctm.18-0154 31104357 PMC6708070

[B30] McLaughlin K. A. Levin M. (2018). Bioelectric signaling in regeneration: mechanisms of ionic controls of growth and form. Dev. Biol. 433 (2), 177–189. 10.1016/j.ydbio.2017.08.032 29291972 PMC5753428

[B31] Morokuma J. Blackiston D. Adams D. S. Seebohm G. Trimmer B. Levin M. (2008). Modulation of potassium channel function confers a hyperproliferative invasive phenotype on embryonic stem cells. Proc. Natl. Acad. Sci. U. S. A. 105 (43), 16608–16613. 10.1073/pnas.0808328105 18931301 PMC2575467

[B32] Nath S. C. Menendez L. Friedrich Ben-Nun I. (2023). Overcoming the variability of iPSCs in the manufacturing of cell-based therapies. Int. J. Mol. Sci. 24 (23). 10.3390/ijms242316929 PMC10706975 38069252

[B33] Nori S. Okada Y. Yasuda A. Tsuji O. Takahashi Y. Kobayashi Y. (2011). Grafted human-induced pluripotent stem-cell-derived neurospheres promote motor functional recovery after spinal cord injury in mice. Proc. Natl. Acad. Sci. U. S. A. 108 (40), 16825–16830. 10.1073/pnas.1108077108 21949375 PMC3189018

[B34] Okano H. Kawahara H. Toriya M. Nakao K. Shibata S. Imai T. (2005). Function of RNA-binding protein Musashi-1 in stem cells. Exp. Cell Res. 306 (2), 349–356. 10.1016/j.yexcr.2005.02.021 15925591

[B35] Pevny L. H. Nicolis S. K. (2010). Sox2 roles in neural stem cells. Int. J. Biochem. Cell Biol. 42 (3), 421–424. 10.1016/j.biocel.2009.08.018 19733254

[B36] Pollard S. M. Conti L. Sun Y. Goffredo D. Smith A. (2006). Adherent neural stem (NS) cells from fetal and adult forebrain. Cereb. Cortex. 16 (Suppl 1), i112–i120. 10.1093/cercor/bhj167 16766697

[B37] Profico D. C. Gelati M. Ferrari D. Sgaravizzi G. Ricciolini C. Projetti Pensi M. (2022). Human neural stem cell-based drug product: clinical and nonclinical characterization. Int. J. Mol. Sci. 23 (21), 13425. 10.3390/ijms232113425 36362211 PMC9653902

[B38] Rapetti-Mauss R. Berenguier C. Allegrini B. Soriani O. (2020). Interplay between ion channels and the Wnt/β-Catenin signaling pathway in cancers. Front. Pharmacol. 11, 525020. 10.3389/fphar.2020.525020 33117152 PMC7552962

[B39] Rosati J. Ferrari D. Altieri F. Tardivo S. Ricciolini C. Fusilli C. (2018). Establishment of stable iPS-derived human neural stem cell lines suitable for cell therapies. Cell Death Dis. 9 (10), 937. 10.1038/s41419-018-0990-2 30224709 PMC6141489

[B40] Schaarschmidt G. Wegner F. Schwarz S. C. Schmidt H. Schwarz J. (2009). Characterization of voltage-gated potassium channels in human neural progenitor cells. PLoS One 4 (7), e6168. 10.1371/journal.pone.0006168 19584922 PMC2702754

[B41] Sempou E. Kostiuk V. Zhu J. Cecilia Guerra M. Tyan L. Hwang W. (2022). Membrane potential drives the exit from pluripotency and cell fate commitment via calcium and mTOR. Nat. Commun. 13 (1), 6681. 10.1038/s41467-022-34363-w 36335122 PMC9637099

[B42] Shetty A. K. Hattiangady B. (2016). Grafted subventricular zone neural stem cells display robust engraftment and similar differentiation properties and form new neurogenic niches in the young and aged hippocampus. Stem Cells Transl. Med. 5 (9), 1204–1215. 10.5966/sctm.2015-0270 27194744 PMC4996439

[B43] Song M. Mohamad O. Chen D. Yu S. P. (2013). Coordinated development of voltage-gated Na+ and K+ currents regulates functional maturation of forebrain neurons derived from human induced pluripotent stem cells. Stem Cells Dev. 22 (10), 1551–1563. 10.1089/scd.2012.0556 23259973 PMC3653388

[B44] Stewart R. R. Zigova T. Luskin M. B. (1999). Potassium currents in precursor cells isolated from the anterior subventricular zone of the neonatal rat forebrain. J. Neurophysiol. 81 (1), 95–102. 10.1152/jn.1999.81.1.95 9914270

[B45] Sugawara K. I. Kurihara H. Negishi M. Saito N. Nakazato Y. Sasaki T. (2002). Nestin as a marker for proliferative endothelium in gliomas. Lab. Investig. 82 (3), 345–351. 10.1038/labinvest.3780428 11896213

[B46] Sun Y. Kong W. Falk A. Hu J. Zhou L. Pollard S. (2009). CD133 (prominin) negative human neural stem cells are clonogenic and tripotent. PLoS One 4 (5), e5498. 10.1371/journal.pone.0005498 19430532 PMC2676510

[B47] Sundelacruz S. Levin M. Kaplan D. L. (2009). Role of membrane potential in the regulation of cell proliferation and differentiation. Stem Cell Rev. Rep. 5 (3), 231–246. 10.1007/s12015-009-9080-2 19562527 PMC10467564

[B48] Talaverón R. Fernández P. Escamilla R. Pastor A. M. Matarredona E. R. Sáez J. C. (2015). Neural progenitor cells isolated from the subventricular zone present hemichannel activity and form functional gap junctions with glial cells. Front. Cell. Neurosci. 9, 411. 10.3389/fncel.2015.00411 26528139 PMC4602088

[B49] Ter P. É. And E. (2010). Molecular background of leak K+ currents: two-pore domain potassium channels. Physiol. Rev. 90 (2), 559–605. 10.1152/physrev.00029.2009 20393194

[B50] Vadlakonda L. Pasupuleti M. Pallu R. (2013). Role of PI3K-AKT-mTOR and wnt signaling pathways in transition of G1-S phase of cell cycle in cancer cells. Front. Oncol. 3, 85. 10.3389/fonc.2013.00085 23596569 PMC3624606

[B51] Verkerke M. Werkman M. H. Donega V. (2025). Stem cell reports review neural stem cells of the subventricular zone: a potential stem cell pool for brain repair in Parkinson’s disease. Stem Cell Rep. 20, 102533. 10.1016/j.stemcr.2025.102533 40513565 PMC12447340

[B52] Vitali I. Fièvre S. Telley L. Oberst P. Bariselli S. Frangeul L. (2018). Progenitor hyperpolarization regulates the sequential generation of neuronal subtypes in the developing neocortex. Cell 174 (5), 1264–1276. 10.1016/j.cell.2018.06.036 30057116 PMC6545245

[B53] Yamada J. Okabe A. Toyoda H. Kilb W. Luhmann H. J. Fukuda A. (2004). Cl-uptake promoting depolarizing GABA actions in immature rat neocortical neurones is mediated by NKCC1. J. Physiol. 557 (Pt 3), 829–841. 10.1113/jphysiol.2004.062471 15090604 PMC1665166

[B54] Zhang J. Zheng X. Wu Z. Wang Y. Chen H. (2025). Neural stem/progenitor cell transplantation therapy for chronic spinal cord injury. J. Neurorestoratology 13 (5), 100223. 10.1016/j.jnrt.2025.100223

[B55] Zhou Y. Wong C. O. Cho K. J. Van Der Hoeven D. Liang H. Thakur D. P. (2015). SIGNAL TRANSDUCTION. Membrane potential modulates plasma membrane phospholipid dynamics and K-Ras signaling. Science 349 (6250), 873–876. 10.1126/science.aaa5619 26293964 PMC4687752

