# Supplementary figures and images for: A conserved bioelectrical signature defines subventricular zone-derived human fetal neural stem cells and tracks their differentiation state
Source: Front Cell Dev Biol. 2026 Mar 10;14:1774119. doi: 10.3389/fcell.2026.1774119 (PMC13014620; doi:10.3389/fcell.2026.1774119)

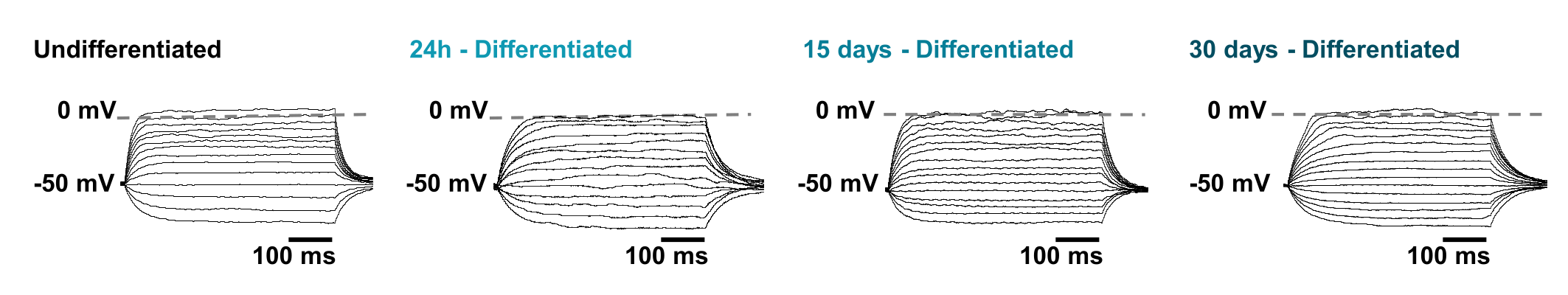

Supplement: Supplementary file 1 [file Image1.tif]
